# Elements of the Practice of Medicine

**Published:** 1840-01-01

**Authors:** 


### ELEMENTS OF THE PRACTICE OF MEDICINE. By *Richard Bright*, M.D. and *Thomas Addison*, M.D. Physicians to Guy's Hospital, and Lecturers on the Practice of Medicine. Vol. I. London, 1839, pp. 613.

"THE authors of the present publication, while engaged in their duties as teachers of the practice of medicine, have frequently felt the want of a work at once elementary and practical to which they might refer their pupils as a companion and assistant during the period of their studies. It is with a view of supplying to themselves such a book of reference, that they undertake to add another to the many elementary works which are already before the public."—*Preface, p.* v.

The volume before us being chiefly intended for the gentlemen composing the practice of physic class at St. Thomas's, whereas our Review is designed to circulate through all classes of the profession, we should be excused for not giving more than a cursory notice of a work confessedly elementary, and confessedly, too, only one of "many elementary works already before the public," and already reviewed in our pages, were it not the fact, that while, nominally, only elementary, it is, in reality, much more.

The present volume is confined to the subject of fever and the phlegmasiæ, and, although, upon the publication of the first fasciculus, the authors proposed to abstain from theoretical discussions, they saw fit, in the second part, to allow even these their place—and thus, instead of a student's companion, of use in the class-room, and in the clinical ward—a lamp for his path, and a lanthorn for his feet, as all elementary works ought to be—we find ourselves called to travel over again, though certainly at improved railway speed, the old roads of long-exploded and universally-rejected theories, the name with which the vague hypotheses of conjectural and imaginative, but unthinking minds are too frequently dignified.

From men so eminently practical as Drs. Bright and Addison are known to be, we shall only be expected to select what they themselves call "sound doctrines and wholesome practice."

One-fourth part of the whole volume is occupied with the subject of (A) FEVER; which our authors treat under the separate heads of Idiopathic, Intermittent, Remittent, Infantile Remittent, and Continued Fevers. This division, it will be seen, completely excludes the synocha or inflammatory fever of Cullen from the list of idiopathic fevers, which, on account of its "extremely rare occurrence, unconnected with some local inflammation or catarrhal affection," is referred to inflammation or catarrh.

### Idiopathic Fever.

With the symptoms and divisions of idiopathic fever our readers are familiar. The only exciting causes mentioned by the authors of the present volume are marsh miasmata and contagion. The former is undoubtedly a principal cause.

### I. Intermitting Fever.

We select a couple of passages on, first—

*Complicated Ague.* "By complicated ague is meant, an ague, to the ordinary phenomena of which, is superadded some unusual local affection, being most commonly seated in the abdomen, in the chest, or within the head. The abdominal complication is most frequent, and in this country is most prevalent during Summer and Autumn. It may not amount to more than that sickness at stomach, and moderate degree of purging, to which many persons in ordinary health are subject at these periods of the year, depending upon the presence of vitiated secretions, especially from the liver. In other instances, however, we have more decided indications of actual inflammation, affecting either the mucous lining of the alimentary canal, the liver, or the spleen, as shown by the presence of the usual symptoms of these respective diseases."

"Thoracic Affections are most prevalent in Winter and Spring. They are usually of an inflammatory character, and present the ordinary signs of bronchitis, of pleurisy, or of pneumonia.

With respect to the head, symptoms of irritation or of inflammation of the brain, or its membranes, have not unfrequently been observed to supervene in the progress of an ague, the patient being affected with violent headache, delirium, stupor, coma, convulsions, or rigid spasm."

And, 2ndly, on—

*Irregular Ague.*—Ague "sometimes changes its type, the quotidian becoming a tertian, and the latter passing into a quartan, or *vice versâ.* In other instances, the disease maintains no determined type whatever, but is constantly varying, when it is said to be erratic. In some cases, the cold stage has been altogether wanting, in others there has been no hot stage, whilst in others there have been a cold and a hot, but no sweating stage. Occasionally the disease is very imperfectly developed, so that the patient only experiences frequent but irregular chills, and a general feeling of indisposition; this is vulgarly expressed by saying that such a person does not shake out, or that he is affected with the *dumb* or *dead* ague. It has also been observed, that the consequences of exposure to miasmata may simulate, or at least impart a periodic character to other diseases, such as neuralgia, headache, or even some forms of actual inflammation." 37, 38.

Passing over the Predisposing and Exciting Causes of Ague; and the Diagnosis and Prognosis of the Disease, when it occurs; we hasten to examine the mode of treatment recommended.

Change of place—emetics—opium in large doses—and the application of tourniquets, to anticipate the expected paroxysm, and break the habit of recurrence so characteristic in all the forms of regular ague, are the means enjoined for the general treatment of the disease. In the cold stage, when the paroxysm has begun, an emetic at the very commencement, or an opiate at any period of its progress, will be found of advantage: and so will the tepid bath; bottles of hot water applied to the feet and stomach; and the free use of warm diluents, or even vinous drinks. In the hot stages, the temperature should be diminished; the clothing of the bed and body lessened; cool, acidulated, and saline drinks, substituted for the warm and stimulating potions which were called for in the previous stage; and opium administered, unless contra-indicated by obvious congestion or inflammation. Of venesection, diaphoretics, and purgatives, our authors speak slightingly. While of another favourite remedy this is all the mention. "Some have recommended cold sponging either with water or vinegar and water, and others the tepid or cold affusion." Cæsar himself, as Cassius marked him when the fit was on him, could not have expressed himself more chillingly. In the sweating stage, tepid diluent drinks are all that is needed, common sense dictating the avoidance of every thing calculated to impede the perspiration. The indication during the period of intermission, is, to cure the disease by preventing the recurrence of the paroxysm. This may be effected either by cinchona or arsenic. The former, given in divided doses of the sulphate of quinine, during the entire intermission; the latter, in combination with laudanum, in doses of from four to six minims of the liq. arsenicalis, three or four times a day, and this will be assisted by the infusion of cascarilla, which forms as convenient a vehicle as any for its administration. "The treatment of irregular, is the same as that of regular ague." In the complicated variety, the complication indicates what modification is necessary in the treatment.

### II. REMITTENT FEVER.

This class of fevers is closely allied to the intermittent, having the same origin and in many respects the same phenomena; and requiring analogous treatment; its most marked difference being clearly pointed out by its name. The paroxysms are less marked, the interval between them witnessing no intermission of symptoms, but only, and that not always, a decided remission. In malignant cases it corresponds so closely to typhus or continued fever, as to be with difficulty only distinguished from it.

" When, in a common continued fever, the mucous membrane of the ilium is considerably involved, that disease often puts on a good deal of a remittent, or rather an irregularly hectic form ; the patient experiencing frequent exacerbations and remissions, not perhaps preceded by chilliness or succeeded by much sweating, yet sufficiently well marked." 55, 56.

" The indications" of treatment " are, first, to subdue or remove that general febrile excitement which prevents the intermission, and thereby converts into a remittent what would otherwise prove an intermittent; and, secondly, to complete the cure, according to the same principles as apply to intermittents."

The first of these indications is to be fulfilled by active purgation at the commencement, or, when the disease at its commencement is accompanied with purging, by mild laxatives to evacuate the contents of the bowels, combined with opiates to allay the irritation of the intestinal canal;—by blood-letting, general—to subdue the violence of the general fever—and topical, where there are symptoms of local congestion or inflammation ;—by saline diaphoretics ; an antiphlogistic regimen ; and mucilaginous drinks.

In pursuance of the second indication, the sulphate of quinine, or, perhaps, the arsenical solution, may be had recourse to. Upon the use of the former in aged persons, there is a practical observation of great value, which we give in the words of the authors.

" When the disease occurs in old people, and in those of impaired constitution, it will very often not only be safe but necessary to have recourse to it as soon as we have succeeded to some extent in subduing the general excitement, and in relieving individual organs, notwithstanding the continuance of symptoms which in a common continued fever are regarded as contra-indicating its use. Under the circumstances mentioned, a moderate heat of skin and frequency of pulse, some degree of headache, and a foul, loaded, or even dry and brown tongue, ought rather to encourage than deter us from the cautious exhibition of the drug; all these symptoms frequently being observed speedily to subside under its use, and thereby furnishing a sufficient evidence of the pathological difference between a marsh and a common continued fever, how much soever they may correspond in their external aspect." 61, 62.

### III. Infantile Remittent Fever.

The description of this disease, as it occurs in children of from one to eight or ten years of age, is highly graphic. As one of the best portions of the volume before us, we have the less hesitation in transcribing it entire; our own experience helping us to verify it in all its parts.

" When a child of from one to eight or ten years of age has a pale, muddy, and dejected or anxious countenance ; a foul tongue ; a tumid belly ; an irregular appetite, and a morbid state of the bowels ; when it picks its nose, and is fretful and restless when awake, and whines, moans, or starts in its sleep, that child is vulgarly said to be suffering from *worms*. If, together with the above symptoms, the child experience occasional accessions of febrile excitement, it is in like manner said to be the subject of *worm-fever*.

These terms, familiar to the unprofessional public, have, as usual, their foundation in correct observation, the diseased conditions alluded to being uniformly associated with derangement of the primæ viæ, and not unfrequently with worms. By the profession, the accompanying fever has been designated *gastric*, or more commonly infantile remittent fever.

With or without fever, the disease is one of extremely frequent occurrence, and moreover one of vast importance, in consequence of the powerful influence which greater or less degrees of it have in modifying, or in actually inducing, a very large proportion of the disorders incident to early life.

The drooping and listlessness of the child, the muddy or pale face, the languid expression, the dark areola around the eyes, and the dilated or unsteady pupil, at once arrest the attention of the observant practitioner, who, on inquiry, is usually informed that the child, without any assignable cause, began to droop and waste in flesh; that its appetite is impaired, voracious, or capricious; that it occasionally complains of pain in its stomach or head; and that the alvine discharges are offensive and of a dark, pale, green, slate, or clay colour; that the child moans, starts, or grinds its teeth during the night; and that it picks its nose during the day. On examination, the tongue is usually found coated with a white mucus, most considerable towards the base; the belly more or less distended; and the breath nauseous or otherwise offensive.

This state of disease may continue for an indefinite period, with the effect of merely occasioning more or less emaciation and loss of healthy complexion, and may be gradually removed either by the voluntary efforts of the constitution, or by the timely interference of art.

Should these symptoms, however, be disregarded, it very often happens that the child begins to experience irregular exacerbation of febrile excitement, often leading the parents to declare that the child is at one time pale with cold, at another burnt up with fever and parched with thirst.

The febrile exacerbations vary much in their intensity, frequency, and duration. There may be one, two, or even three, in the course of the 24 hours; that in the evening being commonly the most severe.

Each exacerbation is marked by restlessness and impatience of any disturbance; a hot and dry skin; a flush on one or both cheeks, especially in those who have a fair and delicate skin; remarkable drowsiness; some hurry of respiration; feebleness of the voice; a short hacking cough; a frequent pulse; more or less thirst; and picking of the nose, lips, tongue, or some other part of the surface, these symptoms varying both in number and degree in different cases. On the subsidence of the paroxysm, the skin cools and relaxes, but in general without much perspiration; the urine deposits a sediment; universal relief succeeds; and the child is left comparatively free from bodily suffering or mental dejection.

If, however, the disease be allowed to proceed uncontrolled, the exacerbations are liable to become more and more severe, and the remissions less distinct; the appetite is entirely lost; the thirst becomes incessant; the tongue gets dry and brown; the lips and teeth covered with a dark sticky mucus; the voice husky or suppressed; the hacking cough more distressing; delirium supervenes; and the debility and emaciation increase, till the patient sinks completely exhausted; or, symptoms of hydrocephalus supervening, coma or convulsions more abruptly close the scene.

For the convenience of description, the symptoms enumerated may, with some propriety, be divided into three groups or orders: the first, characterizing cases unattended by any febrile exacerbation whatever; the second, cases with moderate but distinct febrile exacerbation; and the third, cases of great severity, in which the exacerbations are so acute, and the remissions so imperfect, that the disease approaches the character of a continued fever." pp. 62, 3, 4.

The treatment consists in emptying the intestines of every thing irritating; in correcting the vitiated secretions; and in the due regulation of regimen and diet.

After the morbid condition of the intestines has been to a certain extent removed, the occasional use of the warm-bath will materially contribute to produce a state of convalescence; and that state will be still further promoted by the cautious exhibition of mild, bitter tonics, combined with small doses of the carbonate of soda. In some instances, the sulphate of quinine, in others the chalybeates prove very beneficial.

### IV. Continued Fever.

Under this title, as the least ambiguous, Drs. Bright and Addison comprehend every variety of disease described by other authors as synochus, typhus, adynamic fever, nervous fever, ataxic fever, jail fever, malignant fever, spotted fever, putrid and contagious fevers.

They treat of it under its mild, severe, and complicated forms. Under the complicated form we have it combined with predominant cerebral, abdominal, or thoracic affection; each of which varieties is sketched with a masterly hand.

The indications of treatment are two:—

1st. To cut short the disease, if practicable, by a brisk emetic, active purgation, and one free blood-letting at the commencement, followed up by the cold affusion, or, where the attendants revolt from that remedy, by sponging the surface with cold water or vinegar and water, or vinegar and water with the addition of æther; and by the administration of saline diaphoretic medicines.

2nd. Failing to fulfil the first, to conduct the disease to a successful termination; which is to be accomplished by the judicious employment of occasional laxatives, and of different preparations of mercury, according to the peculiarities of special cases; a low diet, antiphlogistic regimen, and acescent drinks; with a diminished temperature and free ventilation.

Should these means fail of fulfilling either indication, and the *mild* run into the *severe* form, depletion and counter-irritation are further indicated, the latter by the constant application of cold to the shaven scalp—assisted by a blister to the nape of the neck, and mercurial purges. If, after all this, the cerebral disturbance and irritation to a considerable extent remain, small and frequent doses of laudanum are recommended—and if, notwithstanding, the powers of life begin to fail, stimulants and tonics end the catalogue of remedies—and only too frequently exhibit the impotence of medicine.

We transcribe another practical caution of value to every practitioner.

"Before proceeding," they say, "to point out the treatment applicable to the more decided inflammatory complications of fever, it must be observed, generally, that although these inflammations are to be treated on common principles, it must never be forgotten that, along with the inflammation, we have to contend with a fever which is at all times of uncertain duration, and which never fails to impair more or less the vital powers of the system; and, consequently, that in adopting depletory measures, we must employ them with extreme caution and with a sparing hand." p. 108.

In the *cerebral complication* which, from the intensity with which the brain is affected, has obtained the name of brain fever, the blood-letting should be not only free in the first instance, but repeated as often as may be necessary, and, when doubt exists of the propriety of carrying general blood-letting further, the abstraction of blood from the neck and temples by cupping are suggested. A shaven crown—and cold head are of course indispensable—the last being effected by evaporating lotions, or a descending column of water made to impinge upon it. Free evacuations of the alvine canal; abstinence from slops; with the exclusion of light and the suppression of sounds, are also to be resorted to.

Upon the use of the strait-waistcoat in these cases, we entirely concur with the writers, that it is " a proceeding which ought never to be adopted, provided the safety of the patient and of his attendants can be secured by more gentle means."

In the *Complication with Abdominal Affection*, where vomiting prevails attended by tenderness at the pit of the stomach—general blood-letting must be more sparingly resorted to—the use of mercurials, especially of calomel, is contra-indicated—topical blood-letting by leeches—counter-irritation by poultices, blisters, or, better, because more promptly efficacious, the application of the liq. ammon. fortiss. over the region of the stomach; and mild emollient laxatives, are the principal remedies to be depended upon. When diarrhœa is present, as frequently happens, a dose of castor oil, combined with a few drops of laudanum will prove of the greatest service.

Such cases are, generally, protracted; and leave, as their sequel, emaciation, sometimes so deplorable that the bones of the hip and sacrum protrude through the skin, and give rise to foul, sloughing ulcers. Dr. Arnott's water-bed is properly enough recommended where it can be procured; but how few fever patients, comparatively, have any bed at all to lie upon! For these, even the alternative of pillows are scarcely procurable. And in such cases we advise the liberal employment of common lead or soap plaister.

Where the *predominant* is a *thoracic affection*—it may be bronchitis—pneumonia—or pleurisy. And very different treatment being required in these cases, the diagnosis cannot be too careful. We cannot do better here than speak our own thoughts in the words of the volume under review:—" if general blood-letting to any considerable extent be an ambiguous remedy, even in idiopathic bronchitis, it is a practice which requires much more scrupulous caution in fever," (complicated with bronchitis.) " In truth it is rarely admissible; local depletion, by cupping or leeching, or the application of a blister, being much more safe and often sufficiently effective." p. 113.

Where pneumonia supervenes, or pleurisy, the practitioner need have no fear of the lancet, it is his sheet anchor.

In bronchitis—mercury is generally inadmissible—antimony, and the best preparation of it is the tartar emetic of the Pharmacopœia, is *the* remedy to be relied on.

In pneumonia and pleurisy, on the contrary—not antimony, but mercury, is the medicine to be trusted.

With theories, we promised our readers at the commencement of this article, they should not be troubled; and we shall therefore pass by in silence the Authors' Theory of Fever.

We proceed to glance at the contents of the second division of the volume which treats of—

### (B). INFLAMMATION.

After premising a few comments on the ordinary signs of inflammation it proceeds to point out the changes which take place, and the constitutional effects produced by it. The former are—1st. Loss of Cohesion. 2d. Effusion of Serum. 3rd. Effusion of Coagulating Lymph, organizable or coagulable Lymph; inorganizable, or Pus. 4. Arrestment of Organic Function, constituting Gangrene and Sphacelus. The latter are, hectic fever, mortification, ulceration, granulation and cicatrisation; but whether the two last are to be considered as changes at all, appears to us very questionable.

The particular inflammations treated of are, catarrh; influenza; bronchitis; laryngitis; croup; hooping-cough; pneumonia; pneumothorax; pleuritis; phthisis pulmonalis; pericarditis; phrenitis; arachnitis; cerebritis: otitis; cynanche tonsillaris, chronica, pharyngea, membranacea, and parotidea; acute, puerperal, chronic, and latent peritonitis; acute and chronic gastritis; acute and chronic enteritis; inflammation of the cæcum and appendix vermiformis; hepatitis; acute hepatitis, (1st of serous covering, and 2d, of parenchyma); subacute and chronic hepatitis; splenitis, (1st, of serous membrane, 2d, of parenchyma,); nephritis, cystitis, rheumatism and gout.

In noticing the inflammatory affections, as cursorily as our limits require, we shall group them in orders, according to their organic seats; not having space to consider them separately.

### I. Inflammations of the Mucous Membranes.

These may be divided into—1st. *Inflammations of the Mucous Membrane lining the Air-passages,* and 2ndly, *Inflammations of the Mucous Membrane lining the Alimentary Canal.* And these again may be subdivided, the first into Catarrh and Influenza, Laryngitis, Croup, Bronchitis, and Hooping-cough, which are all modifications and degrees of one common disease—calling for the same general treatment, only modified by the specific varieties of the disorder; the second, into Cynanche, Gastritis, Enteritis, or Muco-enteritis, and Cystitis.

*Catarrh.*—"In its most exquisite form, may be said to consist of an inflammatory condition of the mucous membrane of the nose, eyes, frontal sinuses, fauces, trachea, and bronchial tubes; generally attended with pyrexia; for the most part sporadic; but occasionally epidemic, when it receives the name of,"

*Influenza:*—the chief peculiarities of which are, its almost universality; the abruptness of the attack;—and the rapidity with which it increases in severity.

*Laryngitis,* is inflammation of the same membrane where it lines the larynx; and

*Croup* and *Bronchitis* are still inflammations of the same tissue, changed only in their locality, and every variety of symptom by which they are severally distinguished being produced by the altered circumstances of their respective localities. Whereas, hooping, or whooping cough, appears to be an assemblage of all the above, commencing with catarrh, and ending in whoop.

The treatment accordingly, in all these diseases, has certain indications in common, requiring to be fulfilled with more or less energy, according to the seat of the disease and the severity of the symptoms. These indications are, to subdue inflammatory action; allay irritation; mitigate the cough; and facilitate expectoration.

In ordinary catarrh, "little more is required than confinement within doors and abstinence for a few days, together with a liberal use of diluent or cooling drinks to promote perspiration." "Should the febrile symptoms run high, should the constriction of the chest be considerable, and especially if the disease show a tendency to pass into pneumonia or pleurisy, blood-letting may be had recourse to with safety and advantage, to an extent regulated by the severity of the attack, and the constitution of the patient."

Gentle cooling laxatives, and antimonial diaphoretics; with a blister perhaps to the chest where its constriction is painful; and the pediluvium every night; will subdue the violence of the attack, and the irritability consequent upon it will be found to yield very generally to small doses of Dover's powder.

In influenza, blood-letting has never been employed to any great extent, and, indeed, "in some instances it has been found inadmissible, from the great and sudden prostration of strength observed to follow its use."

"As there is perhaps no disease, the treatment of which requires greater caution and circumspection than *Bronchitis*, it would be well for the student to fix firmly in his mind, the principles upon which it ought to be conducted. It is true that the disease in all its forms is one of inflammation, and that the activity of the treatment must be to a certain extent regulated by its intensity. *It must however, be borne in mind that it is inflammation of a mucous membrane, over which depleting* measures exert a very partial control; that in spite of every remedy such inflammation will generally continue an indefinite length of time, although in a subdued degree; that the inflammation as long as it lasts, is attended with a morbid secretion which obstructs the bronchi and seriously interferes with the functions of the lungs; and that, in order to free the bronchial tubes from the excessive secretion, the patient has to pass through a longer or shorter period of great exertion, distress, and want of repose. From this it follows, that all remedies calculated greatly to impair the patient's strength, should be employed with the greatest reserve, and that even in the most acute attacks the period of active depletion is likely to be of very short duration." 196.

"As there is some reason for believing that the peculiar inflammatory product which proves so great a source of danger, (in *croup*) results from the kind, rather than from the mere intensity of the inflammation, we ought, in regard to depletion, to be guided more by the degree of febrile excitement present and by the age and constitutional powers of the patient, than by the mere obstruction to the breathing." 212.

"To be successful, the treatment of acute laryngitis must be prompt and active. As the disease is one of inflammation accompanied with a remarkable tendency to spasm about the glottis, our object should be, to subdue the inflammation by antiphlogistic measures; and allay or prevent the spasm by the simultaneous employment of anodynes, and by the local application of warmth and moisture. If there be any very considerable febrile disturbance, the necessity of general blood-letting is sufficiently apparent; and even if not, should the local affection prove severe, and provided the patient be of good constitution, it ought never to be neglected in the first instance. The patient may be bled to such an extent as to induce a tendency to syncope; immediately after which, two or three grains of calomel, with a grain of opium and a quarter of a grain of tartar emetic, may be given, and repeated every three or four hours, till the constitution becomes affected by it. After general bleeding, 6, 8, 10 or 20 leeches, according to the age of the patient and particular circumstances of the case, may be applied to the external throat, followed either by a large warm poultice, or assiduous sponging with very hot water, &c." 205.

"When consulted very early, even in slight cases, (of *hooping-cough*), it will always be prudent and safe on the part of the practitioner, to adopt antiphlogistic measures, with a view to counteract the tendency to bronchitic and pneumonic inflammation." 220.

Of the other remedies to be employed in these different yet similar diseases, a brief enumeration will suffice:—gentle laxatives rather than drastic purgatives; saline and antimonial diaphoretics; counter-irritants; where considerable irritation is a concomitant, anodynes; and where opiates are inadmissible, camphor and hyoscyamus prove generally efficacious.

As in inflammations of the mucous membrane lining the air-passages, so, in inflammations of the same membrane where it is prolonged over the interior of the alimentary canal, &c. the indications of treatment are:—to subdue inflammation, to allay irritation,—and these will be effected here, as elsewhere, by diminishing the activity of the circulation in the part affected, either by moderate general blood-letting; free topical bleeding; counter-irritants; gentle evacuants rather than active purgatives; diaphoretics, and nauseating ptisans—with the use of the warm-bath, and the cautious administration of anodynes. In acute gastritis, and in acute enteritis, laxative enemata must be had recourse to, and total abstinence from food enjoined. As the former disease subsides, a few grains of carbonate of magnesia, with a few drops of vinum opii, or of the hydrocyanic acid, will allay the irritability of the organ.

In *Enteritis,* "the irritability of the bowels may sometimes be allayed by injecting about three parts of a pint of thin tepid gruel or barley water into the rectum; or still more certainly, by throwing up two or three ozs. of thin starch with fifteen to twenty minims of laudanum, or half an ounce of syrup of poppies," 486.

In *Cystitis*:—"The indications of treatment are, first, to remove if possible the exciting cause, if any exist at the time; secondly, to subdue the inflammation; thirdly, to allay the irritability of the bladder, so as to enable the patient to desist from frequent straining; and lastly, to remove any remaining irritation of the mucous membrane of the urinary passages generally." 557.

### II. Inflammations of the Serous Membranes.

The natural division of these is according to the cavities which they form —to wit, the cerebral, the thoracic, the abdominal, and the articular.

The first will embrace phrenitis and arachnitis; the second, pleuritis and pericarditis; and the third, peritonitis, sero-hepatitis, sero-splenitis, and sero-enteritis.

For the descriptions and pathology of these diseases we must again refer to the work itself—our object is to lay before our readers the principles of practice in their treatment.

In *Phrenitis* and *Arachnitis*, the prompter the measures, and the bolder the treatment, the more successful will be the issue; here, delays are indeed dangerous, whatever is done must be done quickly. Bleeding to syncope—repeated—and again repeated. Leeches. Cupping. A blister to the nape of the neck. Ice or evaporating lotions to the scalp, which should be *shaved* in the very first instance; mercurial inunction rubbed well in, and even used as a dressing for the blister; and calomel pushed by small and frequent doses, to affect the constitution and produce ptyalism—are the leading points of practice in a set of diseases which, if we do not nip them in the bud, will speedily kill our patients.

In *Pleuritis* again, "the treatment is extremely simple; the most successful practice consisting in active depletion and a free use of mercury, so as to produce its specific effects upon the system." 274.

In *Pericarditis*:—"The indications are, to put a stop to the inflammation, and to prevent or arrest effusion and consequent adhesion. These objects are to be accomplished by general and local depletion, and by speedily inducing mercurial action on the system." 325.

In *Peritonitis*:—"The chief part of the treatment consists in general and local depletion, and as speedily as possible bringing the system under the influence of mercury." 427.

And so on, in *every* case of an inflamed serous membrane. Attention to these two principles will simplify the study of inflammatory diseases whether in books, or far better, at the bed-side, and very materially simplify their treatment. In inflammatory affections of the serous membranes, mercury is indicated; blood-letting, general and topical, is indispensable. In inflammatory affections of the mucous membranes, general blood-letting and mercury both, are contra-indicated—topical blood-letting is mainly admissible—antimony in some is essential to success.

We come now to notice, in the last place, a third group of inflammatory diseases, viz.:—

### III. INFLAMMATIONS OF THE PARENCHYMA OF ORGANS.

Under this head are included, cerebritis, pneumonia, phthisis, carditis, hepatitis, splenitis, nephritis, rheumatism and gout.

In *Cerebritis*, the diagnosis is most obscure, and if not made out at the beginning, the error is almost irreparable; the prognosis is most unfavourable, and unless the treatment be most active from the commencement, the case will prove hopeless. Depletion and counter-irritation, are the principal means of subduing the morbid action. The lowest diet and the utmost quiet are indispensable.

In *Pneumonia*, particularly in its truly simple form, the diagnosis is not unattended with difficulty, even to the stethoscopist, "from the absence of those functional signs which he has been taught to regard as characteristic of the least complicated form of the complaint." And to those who are not in the habit of employing the stethoscope the difficulty is increased, from either the very slight degree in which cough or expectoration is present, or their total absence. Upon one diagnostic symptom our authors must be suffered to speak for themselves, as it were impossible for any others to speak better than they do :—

"Of all the symptoms of pneumonia, the most constant and conclusive in a diagnostic point of view is a pungent heat of the surface ; by this symptom alone the first stage of pneumonia may in most instances be readily recognized ; by this symptom alone pneumonia has been repeatedly pronounced to exist, before asking a single question, or making the slightest stethoscopic examination of the chest. The presence of this symptom will seldom mislead even in the most complicated forms of inflammation within the chest. It is by no means contended that it is necessarily present at some period of every case, although that is not improbable; but it may be safely affirmed that when inflammation is confined to the chest, however varied may be the tissues involved in the inflammatory process, provided this symptom be present, pneumonia may be confidently pronounced to form a part in nineteen cases out of twenty, and perhaps in a larger proportion. A similar pungent heat of the surface is now and then observed in certain forms of renal dropsy ; more frequently in continued fever, especially in children ; and still more commonly in the eruptive fevers of the exanthemata and erysipelas ; and as such cases may supervene upon already existing disease within the chest, the fact ought to be carefully remembered." 241-2.

"The treatment must be purely and promptly antiphlogistic." Bleeding to syncope, and mercury to ptyalism, are recommended by one class of practitioners ; the antimonial or contra-stimulant treatment by another. In proportion as the seat of the disease is accurately defined to be either the substance or the lining membrane of the lungs, the indication will be determinate whether the mercurial or the antimonial should be the treatment employed.

We have no room for entering upon the subject of *Phthisis* here. To do justice to the very valuable practical remarks of the authors, would require nearly the whole of the space allotted to the present article. Although peculiarly an English disease, it has baffled every English practitioner, and even in this our day continues to be mistaken in its incipient, and misconducted in its subsequent stages. Indeed, it is only a few weeks since our attention was called to a case where a practitioner who enjoys to a considerable extent the confidence of the public in his own neighbourhood, has mistaken the disease altogether—pronounced the colliquative sweats accompanying it salutary ; and actually poured in medicine with a view to encourage them, until the fears of the family were roused by the *originality* of the practice, for other members of it had passed through the disease to their graves—and another practitioner was called in, to behold the victim die.

Of the treatment of phthisis, all that can be said is well said in the present volume.

"Little as we can place confidence in medicine, and impossible as it is to speak of remedies as calculated to cure the advanced disease, it is pleasing to feel persuaded that when our preventive means have failed, or when they have been too long neglected and the disease has apparently begun, we can often retard its progress, and occasionally have reason to believe that a cure has been effected." 307.

In *Hepatitis*, the characteristic feature of inflammation attacking the parenchyma of organs, obscurity, is present to render difficult the diagnosis. The importance of a correct diagnosis in order to a prognosis which time will verify, must have been felt by every practitioner of even moderate experience. The treatment is obvious—it should be depletory, counter-irritant, mercurial, in a word—antiphlogistic; and " should be marked by all the energy which destructive action, set up in one of the most important organs of the body, must necessarily demand."

In *Splenitis*, the diagnosis is almost always obscure, the prognosis generally favourable—the indication of treatment the same as in other diseases of the same order. Antiphlogistic remedies, promptly and freely employed, " and perhaps a somewhat more cautious use of mercurials than in cases of membranous inflammation," will seldom fail to subdue the violence of the attack, and prepare the way for the administration of tonics to sustain the sinking, or restore the diminished strength of body which follows the attack.

*Nephritis*, " as a purely idiopathic disease, is less frequently met with than most of the other phlegmasiæ, but is of frequent occurrence as an effect of certain acrid substances taken into the stomach, and of calculous matter formed and lodged in the interior of the organ." p. 535.

The well-earned reputation of Dr. Bright, as an accurate and original observer of certain morbid changes in the structure of the kidney, and of certain previously unsuspected connections between diseases of that organ, and an important series of affections, its influence upon which had been previously unsuspected; is amply sustained by the disquisition in the present volume, on inflammation of the above viscus.

The obscurity of its diagnosis; its effect upon the nervous system; and its morbid influence upon the cerebrum itself, are graphically described.

As respects the prognosis, he well remarks that:—

" *The least alarming often proves the most dangerous form of the disorder;* and hence, when it makes its approach insidiously, with little or perhaps no local pain whatever, but with symptoms of oppression of the brain, it is at all times attended with extreme danger, and will, for the most part, be found to prove fatal; and, indeed, whatever may be the form of the complaint, provided the brain become involved, the most serious apprehensions ought at all times to be entertained as to the result; especially if the secretion of urine be not only diminished, but altogether suppressed." p. 546.

The treatment indicated is depletory, and more or less mercurial.

If we were asked to point out the modification called for in the treatment of inflammation when, instead of attacking the membranous tissue, it invades the parenchyma of an organ, we should refer to the tendency which there always is, in the latter case, to a termination in suppuration—as limiting the use both of the lancet and of mercury. And we may say, in conclusion, of the three groups into which we have gathered the inflammations treated of in the present volume:—That—in inflammations of the mucous membranes, the termination is generally in resolution—in those of the serous membranes, in adhesion—in those of the parenchymata, in suppuration. The diagnosis of the three sets of diseases is least difficult in the second, more so in the first—and most of all, in the third. The prognosis is most generally favourable in the first—not so favourable in the second—and still less so in the third. The practice is most decidedly antiphlogistic and depletory in the second—more exclusively antimonial and antistimulant in the first—and in the third partakes of both, without bearing the same amount of either.

We have prolonged this review to such an extent already, as to preclude us from even touching upon Rheumatism and Gout, with the descriptions of which, the present volume concludes.

In bidding its authors a present farewell, we do so with a sincere desire that they will not unnecessarily protract the period which is to elapse before we meet them again in the succeeding portion of their very agreeable work.

That that work will prove a valuable companion, and of real assistance to their numerous pupils, there can be no doubt. That it will prove an acquisition to the library of every practical physician in the land, we likewise believe, or we would not, as we do, heartily recommend it to the attentive perusal of every one of our own readers.

The style in which it is written, without intending a pun upon the names of the writers, is truly chaste and Addisonian—and the practical ideas which that style is employed to convey, are all *bright* and luminous. For ourselves we can only say that we have never tired in its perusal—and rise from its pages with increased respect for its authors.

